# ABO / Rh-D Blood types and susceptibility to Corona Virus Disease-19 in Peshawar, Pakistan

**DOI:** 10.12669/pjms.37.1.3655

**Published:** 2021

**Authors:** Fawad Rahim, Said Amin, Sher Bahadur, Mohammad Noor, Afsheen Mahmood, Huma Gul

**Affiliations:** 1 Dr. Fawad Rahim (FCPS) Medical Teaching Institute / Hayatabad Medical Complex, Peshawar, Pakistan; 2 Dr. Said Amin (FCPS) Medical Teaching Institute / Hayatabad Medical Complex, Peshawar, Pakistan; 3 Dr. Sher Bahadur (MSc. HPM) Medical Teaching Institute / Hayatabad Medical Complex, Peshawar, Pakistan; 4 Dr. Mohammad Noor (FRCPE) Medical Teaching Institute / Hayatabad Medical Complex, Peshawar, Pakistan; 5 Dr. Afsheen Mahmood (FCPS) Medical Teaching Institute / Hayatabad Medical Complex, Peshawar, Pakistan; 6 Dr. Huma Gul (MBBS) Medical Teaching Institute / Hayatabad Medical Complex, Peshawar, Pakistan

**Keywords:** ABO blood group, Rh blood group, disease susceptibility, SARS-CoV-2, COVID-19

## Abstract

**Objectives:**

To determine the association between ABO/Rh-D blood types and susceptibility to SARS-CoV-2 infection in Pakistan.

**Methods:**

In this cross-sectional study, 1935 confirmed cases of COVID-19 were included using consecutive sampling. Age and gender-matched sample of 1935 blood donors was used as a comparison group. Chi-square test and binary logistic regression were used for inferential statistics.

**Results:**

Significantly higher proportion of blood type-B was observed in COVID-19 group (35.9% vs 31.9%, p=0.009). Blood type-AB was found more frequently (14.2% vs 11.8%, p=0.03) in the comparison group. The Rh-D Positive blood types were 93.3% in COVID-19 group and 94.9% in comparison group (p=0.03). The odds of blood type-B, AB and Rh-D positive to test positive for SARS-CoV-2 were 1.195 (95% CI 1.04 – 1.36, p=0.009), 0.80 (95% CI 0.66 – 0.97, p=0.03) and 0.75 (95% CI 0.57- 0.98, p = 0.03), respectively. Blood types A and O did not have significant association with SARS-CoV-2 PCR result (p = 0.22 and 0.88, respectively).

**Conclusions:**

There is significant association between blood types B & AB and susceptibility to COVID-19. There is no association between blood types A and O with COVID-19. Rh- D positive blood types are less susceptible to COVID-19.

## INTRODUCTION

The epidemic of COVID-19 started as an unusual pneumonia in China in December 2019. It quickly spread across the globe & was announced as a pandemic by the World Health Organization.^1^ In terms of human lives, it has affected 40,665,438 & the death tally is 1,121,843 as of 22^nd^ October 2020.^2^ Pakistan reported its first case on 26th February 2020 & till now there are 325,480 confirmed cases & its death tally is 6,702.^3^

The infection severity varies from asymptomatic seroconversion, mild/moderate flu-like illness to cytokine storm resulting in multi-organ failure & death.^4^ Various risk factors for the diverse outcomes have been identified like gender, advanced age, cardiovascular, respiratory and renal disorders.^5^ Molecular abnormalities like distribution of Angiotensin Converting Enzyme (ACE) receptors have been reported to affect susceptibility and outcomes of SARS-CoV-2 infection.^6^

Association of blood types has been found with different communicable and non-communicable diseases.^7^,^8^ It is stated that SARS-CoV-2 is associated with blood type-A.^9^ As reported recently from China by Zhao et al.^9^ that blood group ‘A’ is associated with a higher risk for COVID-19 as compared to other blood groups while blood group ‘O’ offered protection in terms of susceptibility. Zietz et al.^10^ from New York reported susceptibility of blood type-B and protective effect of blood type-O to SARS-CoV-2 infection. Moreover, they also found that Rh-D positive blood groups were linked to higher chances of COVID-19. Same findings have been reported from Massachusetts.^11^

There are contradictory reports regarding the correlation between ABO / Rh-D blood types and vulnerability to COVID-19. To date, there is no published data from Pakistan about the vulnerability of blood types to COVID-19. This issue attracted us to determine the association between ABO/Rh-D blood types and COVID-19 in Peshawar, Pakistan where the ethnicity of the population is fairly homogenous.

## METHODS

This cross-sectional analytical study was conducted at Hayatabad Medical Complex (HMC), Peshawar, Pakistan after review and approval by the institutional review board (IRB) and institutional ethical committee (No. 2184, dated 01-04-2020). All confirmed cases of COVID-19 admitted at HMC were eligible to be included in the study irrespective of their age and gender. The sample size was calculated taking proportions of blood type-A as 32.16% in the normal population and 37.75% in COVID-19 patients,^9^ 95% confidence level, and 3 % margin of error, the sample size was estimated to be 1935 in each group (COVID-19 positive group and comparison group). Using consecutive sampling, 1935 patients with a laboratory-confirmed diagnosis of COVID-19 were included in the study. Age and gender-matched sample of 1935 healthy blood donors from 1^st^ June till 30^th^ December 2019 at the blood bank of HMC was used as a comparison group. Data were collected from admitted patients from 20^th^ April to 31^st^ July 2020. After written informed consent to be included in the study, one ml of blood was collected from COVID-19 patients for blood typing. The sample was analyzed at the hematology laboratory of HMC to determine the blood type as per standard operating procedure. Demographic information, COVID-19 status, and blood type were recorded on a structured proforma.

### Statistical Analysis

The data were analyzed in SPSS version 21. Descriptive statistics for age, gender, and proportions of blood types in both groups were determined. Chi-square test was employed to assess the statistical significance of differences in proportions of blood types between the two groups. P-value of less than 0.05 was taken as significant. Binary logistic regression model was applied to find out the odds of individual blood types and Rh-D type to have positive RT-PCR for SARS-CoV-2.

## RESULTS

The total sample of 3870 (1935 in the COVID-19 group and 1935 in the comparison group) were included in the study. The demographic parameters are shown in Table-I.

**Table-I:** Demographic parameters of the study population.

Parameters	COVID – 19(n=1935)	Comparison group(n=1935)
Age, mean (SD), years	39.73 ± 15.26	32.36 ± 8.65
*Age groups, No. (%)*		
Up to 20 years	147 (7.6%)	194 (10.0%)
21 to 30 years	481 (24.9%)	743 (38.4%)
31 to 40 years	479 (24.8%)	589 (30.4%)
41 to 50 years	338 (17.5%)	398 (20.6%)
Above 50 years	490 (25.3%)	11 (0.6%)
*Gender, No. (%)*		
Male	1328 (68.6%)	1310 (67.7%)
Female	607 (31.4%)	625 (32.3%)

The proportions of blood groups A, B, O, and AB were 27.0%, 35.9%, 25.3%, and 11.8% in the COVID-19 group while these were 28.8%, 31.9%, 25.1% and 14.2% in the comparison group, respectively.

The proportion of blood type-A did not differ significantly in COVID-19 and comparison group (27.0% vs 28.8%, p=0.22). A significantly higher proportion of blood type-B was observed in the COVID-19 group (35.9% vs 31.9%, p=0.009). Approximately the same proportions of blood type-O (25.3% vs 25.1%, p=0.88) were observed in both groups. Blood type-AB was found more frequently (14.2% vs 11.8%) in the comparative sample and was found statistically significant (p=0.03). The proportion of Rh-D Positive blood was higher in the comparison group than in the COVID-19 group (94.9% vs 93.3%, p=0.03).

On logistic regression analysis, the odds of blood type A, B, O, and AB were 0.96 (95% CI 0.79 – 1.05), 1.19 (95% CI 1.04 – 1.36), 1.01 (95% CI 0.87 – 1.16), and 0.80 (95% CI 0.66 – 0.97) to have positive PCR for SARS-CoV-2. Rh-D Positive blood types were less likely (odds = 0.75, 95% CI 0.57- 0.98) to have positive PCR (Table-II).

**Table-II:** The ABO and Rh blood types distribution in COVID-19 group and comparison group.

	*ABO Blood types*			*Rh status*	

	A	B	O	AB	Rh+
COVID-19 group, No. (%)	523 / 1080 (27.0%)	694 / 1311(35.9%)	490 / 976 (25.3%)	228 / 503(11.8%)	1806 /1935 (93.3%)
Comparison group, No. (%)	557 / 1080 (28.8%)	617 / 1311 (31.9%)	486 / 976 (25.1%)	275 / 503(14.2%)	1837 / 1935 (94.9%)
p value	0.22	0.009	0.88	0.03	0.03
Odds to be PCR positive with 95% CI	0.96(0.79 – 1.05)	1.19(1.04 – 1.36)	1.01(0.87 – 1.16)	0.80(0.66 – 0.97)	0.75(0.57- 0.98)

## DISCUSSION

There has been increasing interest to identify patient-specific risk factors that would determine vulnerability to SARS-CoV-2 infection. Apart from demographic and epidemiological risk factors, the role played by biological markers has been studied.^12^-^14^ Of late, ABO blood type and Rhesus status have been suggested as a cause for variable predisposition to COVID-19.^15^ Studies on the relationship of ABO/Rh-D blood types with SARS-CoV-2 infection are not consistent in their findings, and researchers have not arrived at a plausible explanation for their results.

We found a significantly higher proportion of blood type-B in COVID-19 group than in the control group (35.9% and 31.9%, respectively; p=0.009) with higher odds (1.19, 95% CI 1.04 – 1.36) of testing positive. This is consistent with the results reported by Latz et al.^11^ from Boston and Zietz et al.^10^ from New York, USA. Our study is more in line with the study by Zietz et al. probably there was a 10% representation of Asians in their sample population. Gerard et. Al^16^ arrived at a similar conclusion when they analyzed published data relying on the status of Anti-A antibody. Zhao et al.^9^, Li et al.^17^ and Fan et al.^18^ have reported equal representation of blood type-B in COVID-19 and control groups in their studies from Wuhan, China. A similar result has been reported by Abdollahi et al.^19^ This variation from Chinese and Iranian studies may be explained on genetic clustering of susceptible loci which may vary among different ethnicities.^20^ Another reason for the contradictory findings as compared to Zhao et al.^9^, Li et al.^17^ and Fan et al.^18^ may be the homogenous sample of Chinese ethnicity in their studies.

In this study, almost equal proportions of blood type-O were observed in COVID-19 and control groups (25.3% and 25.1%, respectively; p=0.88) with odds of 1.01 (95% CI 0.87 – 1.16). This is in partial agreement with published data which suggests blood type-O have decreased risk of acquiring SARS-CoV-2 infection.^9^-^11^,^16^,^17^,^19^,^21^ This protection has been attributed to Anti-A antibodies in those with blood type-O.^22^ Since people with blood type-B also have Anti-A antibodies like blood type-O, differences in immunoglobulin subclass (IgM in former and IgG in latter) in Anti-A antibodies have been postulated for the consistently documented protective behavior of blood type-O.^16^,^23^

There was nearly equal representation (27.0% and 28.8%; p=0.22) of blood type-A in COVID-19 and control groups. Blood type-A did not have higher odds (0.96, 95 % CI 0.79 – 1.05) of PCR positivity. These findings are similar to the data reported from research conducted in New York, Boston, and Tehran.^10^,^11^,^19^ However, this contradicts the findings of studies from china.^9^,^17^,^18^ and Pakistan.^21^ The reasons for different findings as compared to Zhao et al.^9^, Li et al.^17^, and Fan et al.^18^ may be due to ethnic differences in study populations. Moreover, the study for Pakistan had a relatively smaller sample.

There was a higher proportion of type-AB in comparison group as compared to the COVID-19 group (14.2 % and 11.8%, p=0.03). AB blood type was found to be less vulnerable to SARS-CoV-2 (Odds = 0.80, 95% CI 0.66 – 0.97). These findings contrast with those reported by Zhao et al^9^., Latz et al.^11^ and Abdollahi et al.^19^ We could not explain the reasons for the dichotomy except on ethnic and genetic basis.

The proportion of Rh-positive blood types was more in the comparison group as compared to the COVID-19 group (94.9 and 93.3%, p=0.03). The odds of Rh-positive blood types to be SARS-CoV-2 PCR positive were 0.75 (95% CI 0.57- 0.98). This is in contrast to that reported by Zietz et al.^10^ and Latz et al.^11^ However, Abdollahi et al.^19^ have observed no correlation between Rh(D) blood type and susceptibility to SARS-CoV-2.

Gerard et al.^16^ used data published by Zhao et al.^9^ from Wuhan, China, and analyzed it relying on the status of Anti-A antibodies. It was found that those having Anti-A (blood type- B and O) were less likely to have COVID-19. Latz et al.^11^ and Zietz et al.^10^ reported from the USA that blood type-B is more susceptible to be SARS-CoV-2 positive. Our data reveal that blood group ‘B’ is more likely to get COVID-19. Despite having Anti-A in their serum, people with blood type-B are more prone to SARS-CoV-2. This indicates that the presence of Anti-A antibodies by itself is not the only predictor of predisposition to SARS-CoV-2. The mechanism underlying the potential association between ABO blood type and predisposition to SARS-CoV-2 needs further research.

There is evolving data regarding association of ABO-Rh blood types and susceptibility to SARS-CoV-2 from China and developed western countries. But there is paucity of data from the developing countries and particularly South Asia. This study will add to the international pool of data on association of ABO-Rh blood types and COVID-19. The results of the study might be helpful in risk stratification of healthcare workers / public and to prioritize vaccination for the most vulnerable group.

The strength of the study was its relatively larger and representative homogenous sample in both groups. We used age and gender matched ABO blood type distribution of blood donors as a comparison group which was comparable to the ABO blood type distribution in general population of Peshawar.^24^ It is a single-center experience comprising mainly the Pashtun population of Khyber Pakhtunkhwa province of Pakistan and may not be representative of the entire Pakistani population and this is considered as a study limitation.

## CONCLUSIONS

Blood type-B has a statistically significant higher risk to acquire SARS-CoV-2 infection as compared to blood types-A, O, and AB. Blood types-A and O did not have any significant positive or negative association with the SARS-CoV-2 PCR result. In contrast, blood type-AB offered protection from the infection as it was less likely to test positive as compared to non-AB blood types. Rh-D positive blood types were less susceptible to COVID-19. Some of the results of this study are in contrast with published data from China, the United States, and Iran. We do not have any reasonable explanation for the disagreements except for the differences in ethnicity and genetic background of the study populations. We propose similar work shall be carried out on a larger sample to have a clearer insight into the association between ABO/Rh-D blood types and susceptibility to COVID-19.

### Authors’ contributions:

All authors had full access to all the data in the study and the corresponding author had final responsibility for the decision to submit for publication.

**FR, SA, SB and MN:** Contributed to conceiving the concept and study design.

**AM and HG:** Enrolled patients and acquired and entered the data.

**SB:** Analyzed the data.

**FR, SA, SB and MN**: Prepared the manuscript.

All authors critically reviewed and approved the manuscript for final submission.

**FR**: Is responsible for the accuracy or integrity of the research work.
